# Modeling Commercial Freshwater Turtle Production on US Farms for Pet and Meat Markets

**DOI:** 10.1371/journal.pone.0139053

**Published:** 2015-09-25

**Authors:** Ivana Mali, Hsiao-Hsuan Wang, William E. Grant, Mark Feldman, Michael R. J. Forstner

**Affiliations:** 1 Department of Biology, Texas State University, San Marcos, Texas, United States of America; 2 Department of Wildlife and Fisheries Sciences, Texas A&M University, College Station, Texas, United States of America; 3 PO Box 285, Kerikeri, Northland, New Zealand; Universidad Veracruzana, MEXICO

## Abstract

Freshwater turtles are being exploited for meat, eggs, traditional medicine, and pet trade. As a response, turtle farming became a booming aquaculture industry in the past two decades, specifically in the southeastern states of the United States of America (US) and across Southeast Asia. However, US turtle farms are currently producing turtles only for the pet trade while commercial trappers remain focused on catching the largest individuals from the wild. In our analyses we have created a biological and economic model that describes farming operations on a representative turtle farm in Louisiana. We first modeled current production of hatchling and yearling red-eared slider turtles (*Trachemys scripta elegans*) (i.e., traditional farming) for foreign and domestic pet markets, respectively. We tested the possibility of harvesting adult turtles from the breeding stock for sale to meat markets to enable alternative markets for the farmers, while decreasing the continued pressures on wild populations (i.e., non-traditional farming). Our economic model required current profit requirements of ~$13/turtle or ~$20.31/kg of meat from non-traditional farming in order to acquire the same profit as traditional farming, a value which currently exceeds market values of red-eared sliders. However, increasing competition with Asian turtle farms and decreasing hatchling prices may force the shift in the US toward producing turtles for meat markets. In addition, our model can be modified and applied to more desirable species on the meat market once more knowledge is acquired about species life histories and space requirements under farmed conditions.

## Introduction

Unrestricted commercial harvest of wildlife can lead to species declines and extinction [1, 2], [3]. Chelonians have been exploited for meat, eggs, and traditional medicine since the 16^th^ century [4], and more recently for the pet trade [5–7]. Harvest levels of wild populations are often reported as excessive and unsustainable [8–10]. Asia, China in particular, is leading the world in turtle consumption as food [11–13]. High demands for freshwater turtle meat has put significant pressure on wild populations not only in Asia but also in other regions of the world [8], [14], [15]. For example, many North American species are being targeted to supply depleted Asian food markets [14]. Turtle captive breeding has expanded in the last two decades in China and other Southeast Asian countries to meet this demand [16–18], and freshwater turtle farming became a booming aquaculture business in the southeastern United States of America (US) in the early 1990s (US) [19].

**Fig 1:**
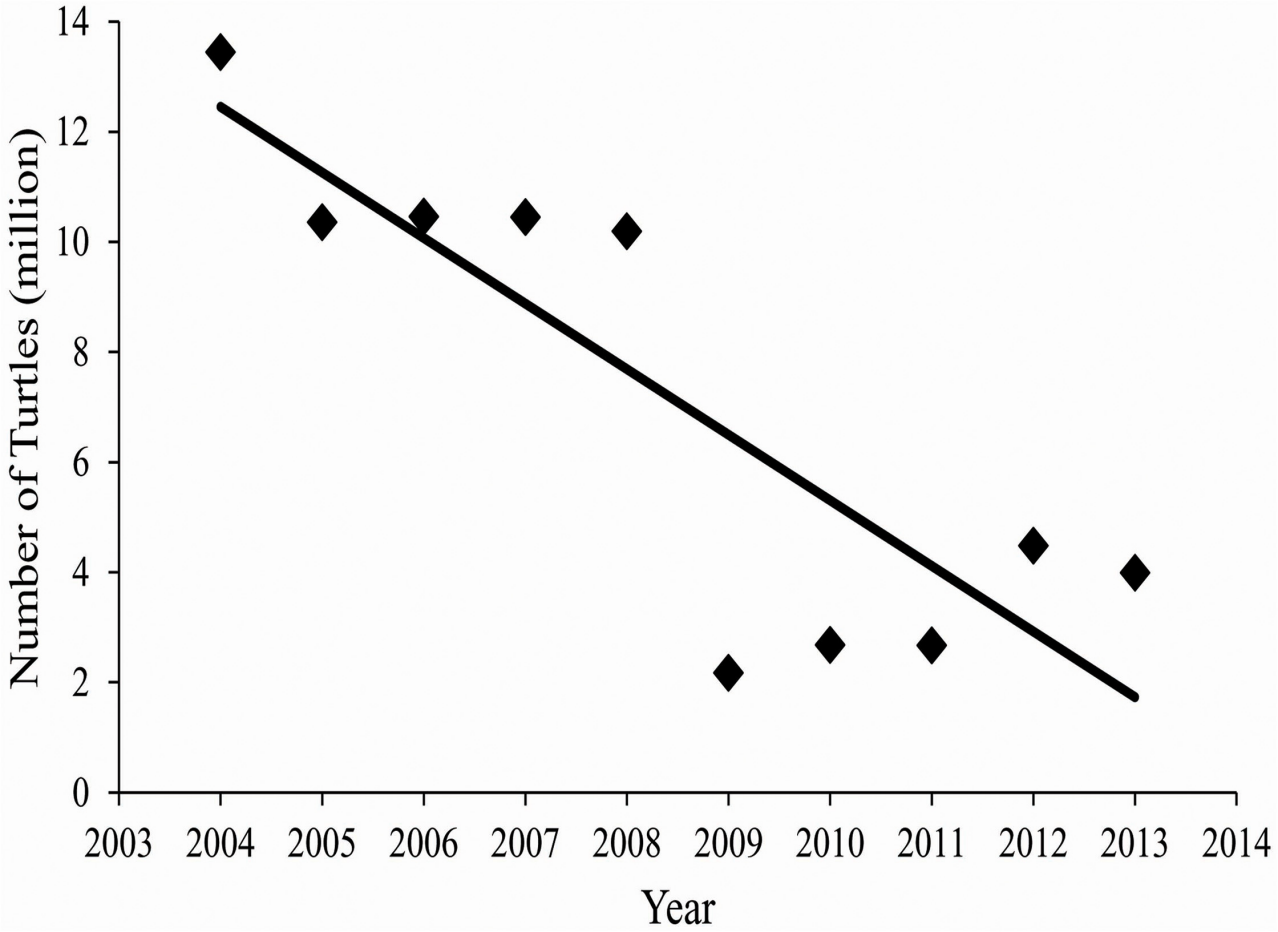
Annual production of turtles on Louisiana turtle farms from 2004–2013 reported by turtle farms to the Louisiana Department of Agriculture.

In the US, Louisiana is the leading freshwater turtle exporter of both wild caught and captive bred individuals [15]. For export to Asian food markets, turtle trappers focus on harvesting the largest individuals from wild populations [20], however, excessive adult harvests is not sustainable and represents a major contribution to declining turtle populations [10]. In contrast to wild turtle trappers, US turtle farmers do not produce adult turtles for profit but rear hatchlings for the pet trade and Asian turtle farms [19] (Jesse Evans, pers. comm.). While newborn hatchlings can be exported to foreign countries, the US restricts selling turtles smaller than 4 inches, 101.6 mm carapace length (CL), as pets because of turtle associated human *Salmonella* infections [21]. Every year, turtle farms bring millions of dollars to the Louisiana economy [22]. However, in the last decade, the total number of turtles produced has decreased from 13.4 million in 2004 to 4 million in 2013 [22] (Fig 1). The Louisiana Department of Agriculture and turtle farmers speculate that the decline is due to lack of demand for hatchling turtles from Asia, where turtle farms are becoming well-established and self-sustained in recent years (Jesse Evans, Concordia Turtle Farm).

Apart from reports on annual hatchling production on farms, there are no models that describe the biological and economic dynamics for turtle farming operation in the US. The future of turtle farming is not only important for the economy of the region, but also has potential to serve as the foundation for conservation and recovery of wild turtle populations. For example, the rapid growth of the salmon aquaculture industry has outcompeted the fishing industry in some regions of the world [23] (e.g., Alaska). More relevant, farming and head-starting of the once endangered American Alligator (*Alligator mississippiensis*) not only enabled its recovery from endangered to delisted, but also enabled the sustainable harvest regimes to be put on wild populations [24].

Hence, the purpose of this study was threefold: 1) to use information and knowledge currently available for freshwater turtle biology under farmed conditions and develop a biological model that describes the dynamics of traditional hatchling and yearling production on a representative farm in Louisiana, 2) to modify this model and test the possibility of harvesting adult turtles from the stock for foreign meat markets in addition to raising and harvesting hatchling and yearling turtles, 3) to develop economic model describing the current and future alternative profits to project the future of turtle farms, and 4) to present Porter five forces analysis [25] to aid in our understanding of general strengths and weaknesses of turtle farming industry.

We used the red-eared slider (*Trachemys scripta elegans*) as a model species. The red-eared slider has a large geographic range in North America [26] and is one of the most commonly farmed freshwater turtle in the US. Although red-eared sliders are still considered common in the wild, declines due to unregulated harvest in the southeast US have been reported [8]. On US farms, red-eared sliders are produced and sold as hatchlings or 4 in turtles (i.e., yearlings); however, adult red-eared sliders can be found on Asian meat markets [27]. For the information that remained unknown (i.e., relationship between female size and her individual reproductive output), we incorporated a few simplifications in these models based on published parameters for wild populations. Therefore, our quantitative results at this stage must remain a tentative approximation.

## Materials and Methods

We visited one of the oldest and most successful turtle farms in the southeast US and requested information about the farm operations, turtle biological parameters, and economical parameters. We obtained no ethics committee/IRB approval as the data was requested and received from the farm on their operations and economics of the operations. There was not any human individual or survey questionnaire administered. IACUC permit was not obtained because we had no interaction with vertebrate animals. As farms are not federally or state dependent, they are not required to obtain IACUC permit.

### Biological Model

**Fig 2:**
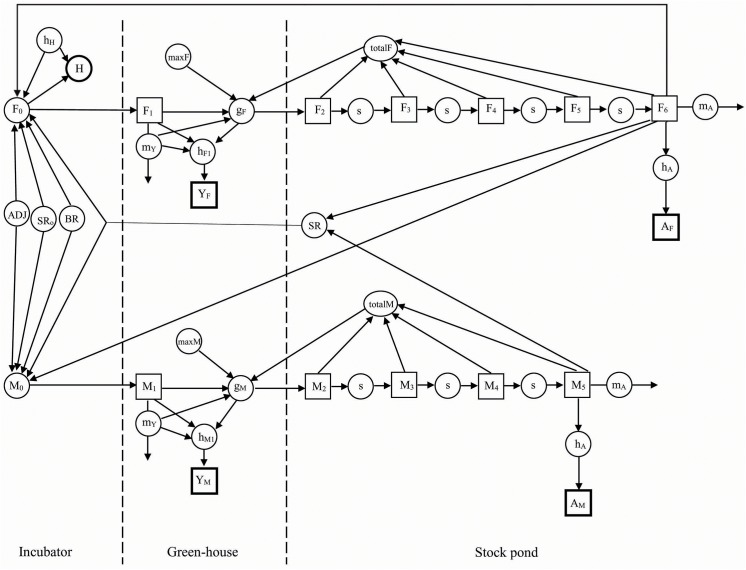
Conceptual diagram of the freshwater turtle farming operations (non-stochastic model). The model was developed for male and female portion of the population. Broken vertical lines separate the operations into egg incubation in closed facilities (Incubator), raising hatchlings to 4 inch turtles or for head-starting in green-house ponds (Green-house), and maintenance of turtles in the outdoor artificial stock ponds (Stock pond). In the model, survival (s), hatchling transfer to the green-house ponds (F_0_ and M_0_), and head-start transfer to the stock pond (g_F_ and g_m_) are illustrated by arrows (material transfers) entering. Mortality (m_Y_ and m_A_) and harvest (H, h_F1_, h_M1_, h_A_) by arrows (material transfers) leaving. Squares (state variables) represent number of males and females of each age class (F_1_–F_6_ and M_1_–F_5_) and the total numbers of yearlings and adults sold over time (Y_F_, Y_M_, A_F_, A_M_). Hatchlings are sold the same year that eggs are laid and the number of hatchlings sold annually is represented by H (circle).

We constructed the model (Fig 2) using STELLA^®^ 7.0.1. graphical dynamic simulation software. We created an age-structured matrix population model for females and for males. The number of age classes depended on the age at maturity for males and females because we grouped all mature individuals into one age class. We defined the time (t) as a one year interval, with hatchlings modeled as being hatched and sold the same year the eggs are laid (i.e., for red-eared sliders eggs hatch two months after laying). The symbols, their definitions, and parameter values are represented in Table 1 while the model is described in text.

**Table 1:** The list of the baseline parameters for development of the population dynamics model for freshwater turtles under farmed conditions and their values for farmed red-eared sliders. Majority of parameter values were acquired either from the personnel on the farms or based on independent experiment while the rest of the parameters were actively managed in the model.

Parameters	Symbol/Equation	Values
Max stock density (per hectare)	*SD*	12,500
Total hectares (stock pond)	*hectares*	2
Max proportion of total females	*p*	0.75
Max number of females	*maxF* = *SD* * *hectares* * *p*	18,750
Max number of males	*maxM* = *SD* * *hectares* * (1 − *p*)	6,250
Female/male stock survivorship	*s*	0.97
Adult stock mortality	*m*_*F*6_ and *m*_*M5*_	0.03
Greenhouse turtle mortality	*m*_*F*1_ and *m*_*M*1_	0.15
Clutch size	*CS*	10 (mean)
Number of clutches per season	*NC*	3
Hatch rate	*HR*	0.85
Per capita birth rate under *SR*_*o*_ ^a^	*BR* = *CS* * *NC* * *HR* * 0.95	24.2 (mean)
Birth adjustment rate	ADJ	managed
Age class (females and males)	*F*_0_ − *F*_6_ and *M*_0_ − *M*_5_	
Optimal adult sex ratio (M:F)	*SR*_*o*_	0.33
True adult sex ratio (M:F)	*SR* = *M*_5_/*F*_6_	
Total number of stock females	*totalF* = *F*_2_ + *F*_3_ + *F*_4_ + *F*_5_ + *F*_6_	
Total number of stock males	*totalM* = *M*_2_ + *M*_3_ + *M*_4_ + *M*_5_	
Hatchling harvest rate	*h*_*H*_	managed
Adult harvest rate	*h*_*A*_	managed

Majority of parameter values were acquired either from the personnel on the farms or based on independent experiment while the rest of the parameters were actively managed in the model.

^a^ For red-eared sliders, 95% of adult females will nest under optimal sex ratio (*SR*_*o*_).

Farms usually consist of a number of artificial ponds containing stock turtles, with enclosed sand beaches surrounding the ponds. Adults are not harvested, but maintained for egg production. We assumed no density dependent growth rates so long that the stock density is equal or less than the threshold. This was derived, in part, from data reported from the farm production data and brood stock maintenance over time. For example, farmers are providing more feed than it can be consumed by the stock, which was evident by many pellets floating unconsumed for days until they are degraded. We defined the optimal sex ratio as the minimum number of sexually mature males to number of sexually mature females required to inseminate majority of adult females in the population. Every year, gravid females nest on the pond beaches, and eggs are collected and incubated in indoor facilities, as is common for all commercial freshwater turtle operations. Average per capita birth rate is the product of average clutch size, number of clutches per season per female, and the hatch rate. Red-eared sliders have temperature dependent sex determination, where higher incubation temperatures produce females [28, 29]. Since eggs are incubated indoors, the sex of the hatchlings can be directly manipulated. Therefore, we created birth rate adjustment parameter, which controls for proportion of female vs male hatchlings produced. This parameter is adjusted so that the majority of eggs are incubated at higher temperatures (i.e., similar to traditional farming), producing females. Small proportion is incubated at lower temperatures in order to repopulate male portion of the stock. Total hatchling production depends on the number of reproductively active females, per capita birth rate, and the adult sex ratio. As long as stock sex ratio is greater or equal than optimal sex ratio, 95% of adult females will lay fertile eggs in a given season. Otherwise, the percentage of fertile eggs will linearly decline.

A portion of the hatchlings are sold shortly after emerging or transferred to green-house ponds until the following year, when yearlings are either sold on domestic pet markets or added to the stock population. In order to keep farms self-sustaining, the yearlings in the model are primarily used to repopulate the stock in order to keep it at maximum allowable stock density, and the surplus is then available to be sold. Because the sex ratio is important in the stock, an additional parameter was created that represents maximum allowable number of females and males in a stock, and their values depend on the maximum stock density and optimal sex ratio. For the stock population, survivorship rates determine the number of individuals entering the next age group.

Under traditional management regimes, stock turtles are not sold on commercial levels and only hatchling and yearling turtles generate revenue. For simplicity of the model, hatchling harvest is only applied to female hatchling portion nt in the stock, an additional parameters was created that until they are degrated.ent best cas(i.e., on pet markets there are no sex preferences for hatchling and yearling turtles). Any un-harvested portion of hatchlings is transferred to the green-house ponds. For yearlings, turtles that remain in green-house ponds, after the stock is repopulated to its threshold density, are sold for profit. To test the possibility and profitability of selling adults (i.e., for meat markets), we later implemented various harvest rates of adults. The implemented equations are presented in Table 2.

**Table 2:** Summary of the dynamics between age classes and turtle production in a conceptual model of freshwater turtle farming.

System of Interest	Conceptual Formula/Equation
Female hatchlings added to the green-house *F*_0_(*t*)	IF (SR(t) ≤ SR_o_) THEN (*F*_6_(t) * BR * ADJ * (1 − *h*_*H*_)) $\mathrm{ELSE}\;(\frac{1}{{\mathrm{SR}}_{o}}*\mathrm{SR}(t)*F_{6}(t)*\mathrm{BR}*\mathrm{ADJ}*(1-h_{H}))$
Female hatchling harvest *H*(*t*)	$F_{0}(t)*h_{H}*(\frac{1}{{1-h}_{H}})$
Male hatchlings added to the green-house *M*_0_(*t*)	IF (SR(t) ≤ SR_o_) THEN (*F*_6_(t) * BR * (1 − ADJ)) $\mathrm{ELSE}\;(\frac{1}{{\mathrm{SR}}_{o}}*\mathrm{SR}(t)*F_{6}(t)*\mathrm{BR}*(1-\mathrm{ADJ}))$
Female head-starts added to the stock *g*_*F*_(*t*)	IF(totalF(t − 1) ≥ maxF)THEN (0)ELSE IF (*F*_1_(t − 1) * (1 − *m*_*Y*_)) ≥ (*maxF* − totalF(t − 1)) THEN (*maxF* − totalF(t − 1)) ELSE (*F*_1_(t − 1) * (1 − *m*_*Y*_)))
Male head-starts added to the stock *g*_*M*_(*t*)	IF(totalM(t − 1) ≥ maxM)THEN (0)ELSE IF (*M*_1_(t − 1) * (1 − *m*_*Y*_)) ≥ (*maxM* − totalM(t − 1)) THEN (*maxM* − totalM(t − 1)) ELSE (*M*_1_(t − 1) * (1 − *m*_*Y*_)))
Female yearling harvest *h*_*F*1_(*t*)	*F*_1_(*t*) − *m*_*Y*_ − *g*_*F*_ (*t*)
Male yearling harvest *h*_*M*1_(*t*)	*M*_1_(*t*) − *m*_*Y*_ − *g*_*M*_ (*t*)
Female stock growth *F*_*i*_(*t*) *(i = 3–6)*	*F*_*i*−1_(t − 1) * s
Male stock growth *M*_*i*_(*t*) *(i = 3–5)*	*M*_*i*−1_(t − 1) * s
Adult harvest *A*_*F*_ and *A*_*M*_	*F*_6_ * *h*_*A*_ and *M*_5_ * *h*_*A*_

### Parameters Values

To put the conceptual model into practice, we used parameter values for farmed red-eared sliders. We obtained values for the baseline parameters by requesting and receiving the data from personnel on a representative turtle farm in Louisiana or by actively managing the parameter values in the model (i.e., birth adjustment rate, optimal sex ratio and stock density). Red-eared sliders stock density can reach the extremes of 37,500 individuals per hectare; however, 12,500 individuals per hectare is a preferred healthy stock density. Therefore, we treat the maximum capacity- threshold at 12,500 turtles per hectare. At our representative farm, a total of ~2 hectares contains red-eared sliders, making a total stock density of 25,000 turtles. Survivorship of the stock is usually high (~0.97), considering that the stock ponds are cleaned every 2–3 years. However, in an event of warm winters, a mass mortality of up to 10% can result, with these events occurring, on average, every six years (Jesse Evans, Concordia Turtle Farm).

Optimal sex ratio for adult turtles is estimated to be 1:3, male:female, which presumably enables 95% of the females to lay fertile eggs. The nesting season lasts from April through August, but the egg collection usually ends in July. Under farmed conditions, females mature between five and six years of age, when they approximately weigh 800g and have 170mm CL. However, mature females can weigh up to 2300g and measure up to 230mm CL. Red-eared sliders are known to produce 3–5 clutches per season and 8–22 eggs per clutch. For farmed red-eared sliders, clutch size is weakly correlated to the size of the adult females (i.e., r^2^ = 0.2). However, the same data shows that across all females, clutch size is normally distributed with mean of 10 ± 2.8 standard deviation (sd). Incubation period is ~60 days, with a hatch rate of 85%. Upon emergence, hatchlings are either sold to foreign markets or kept in green house ponds until the following year, when they reach four inch in size. Turtles that are transferred to greenhouse ponds include not only healthy looking individuals, but also turtles with shell defects due to improper emergence from the shell and turtles with genetic defects. Therefore, despite the fact that the green-house is heated during winter and the turtles are artificially fed, the mean mortality rate approaches 15%. For stock turtles, we created five age classes for females, with all reproductively mature females, classified in one age group. The male portion of the stock was created in a similar manner, but since males mature earlier, we created four age classes, with reproductively mature males in one age class.

To describe traditional farming, we ran simulation models using average parameter values for survivorship and per capita birth rate, non-stochastic model. To test non-traditional farming, we incorporated a range of harvest rates of adult male and female portion of the stock. In all simulations, we manipulated hatchling harvest rate, adult harvest rates, and birth adjustment rate in order to maintain the optimal sex ratio of adults in the stock population (0.33), maximum threshold stock density (25,000 turtles), and to always sell ~55,000 yearling turtles. We managed the constant production of four inch turtles because the market for yearling turtles is more stable than hatchling turtle market, the profit is higher for yearlings, and the farm is selling 50–60k hatchlings annually. We ran 100 year simulations and used the numerical outcomes of the models post initial oscillations, when production stabilized to constant values.

### Bio-economic Model

We created a bio-economic model that includes projections of the expected total costs and expected total revenue associated with farming production. Expected total profit for traditional farming is a function of two major components: total cost and total revenue. Each major component is then broken down to hatchling and yearling costs and revenue. Expected total profit for traditional farming is calculated:

$$E(P_{tr})=\sum_{t=1}^{n}e^{-dt}e^{mt}\;[R(h(t))+R(y(t))]-\sum_{t=1}^{n}e^{-dt}[C(h(t))+C(b(t))]$$

where *d* and *m* are the discount and market rates, respectfully, *n* is the production cycle, and *t* represents the year. *R*(*h*(*t*)) and *R*(*y*(*t*)) represent hatchling and yearling revenue at time *t*, respetivelly. *C*(*h*(*t*)) represents cost of hatchling production, and *C*(*b*(*t*)) represents cost of yearling production including the head-start turtles at time *t*.

Hatchling red-eared slider prices in the past decade have varied from $0.35‒0.50 (mean = 0.41) while the prices for yearlings remained constant at $7.50‒8.00. The production cost per hatchling is currently around $0.25 while the production cost of yearlings is significantly higher, ~$4.5 per capita, due to high heating cost of greenhouse ponds. The costs of raising hatchlings and yearlings include the costs of maintaining the stock and represent close approximations (Jesse Evans, Concordia Turtle Farm). We used the above information to calculate each component of the above equation as follows:

R(h(t))=H(t)*0.35

R(y(t))=Y(t)*7.50

C(h(t))=H(t)*0.25

C(b(t))=B(t)*4.50

where, *H*(*t*) represents the number of hatchlings produced for the foreign market at time *t*, *Y*(*t*) represents the number of yearlings produced for the domestic market at time *t*, and *B*(*t*) represents the number of yearlings produced for both, the market and the head-starting at time *t*.

For the non-traditional model that included 0.4 harvest rate of adult stock for meat market, we calculated the minimum profit requirements per adult turtle, *P*(*a*(*t*)), that would meet the same profit as traditional model at time *t*, using the following equation:

$$P(a(t))={(P}_{tr}(t)-P_{ntr}(t))/A(t)$$

where, *P*_*tr*_(*t*) is profit from traditional farming at time *t*, *P*_*ntr*_(*t*) is profit from non-traditional farming made by selling hatchlings and yearlings at time *t*, and *A*(*t*) is the number of adults harvested for market at time *t*.

### Competitive Forces of Turtle Farming

The Porter five forces analysis is a tool used to understand underlying drivers of a particular industry such as the competitive forces and their underlying causes, as well as the roots of profitability [25]. We assessed the risks associated with the turtle farming industry based on five factors:

1. The potential of new entrants into the industry.
2. Competition in the turtle farming industry.
3. The threat of substitute products.
4. Supplier power.
5. Buyer power.

## Results

### Biology

**Fig 3:**
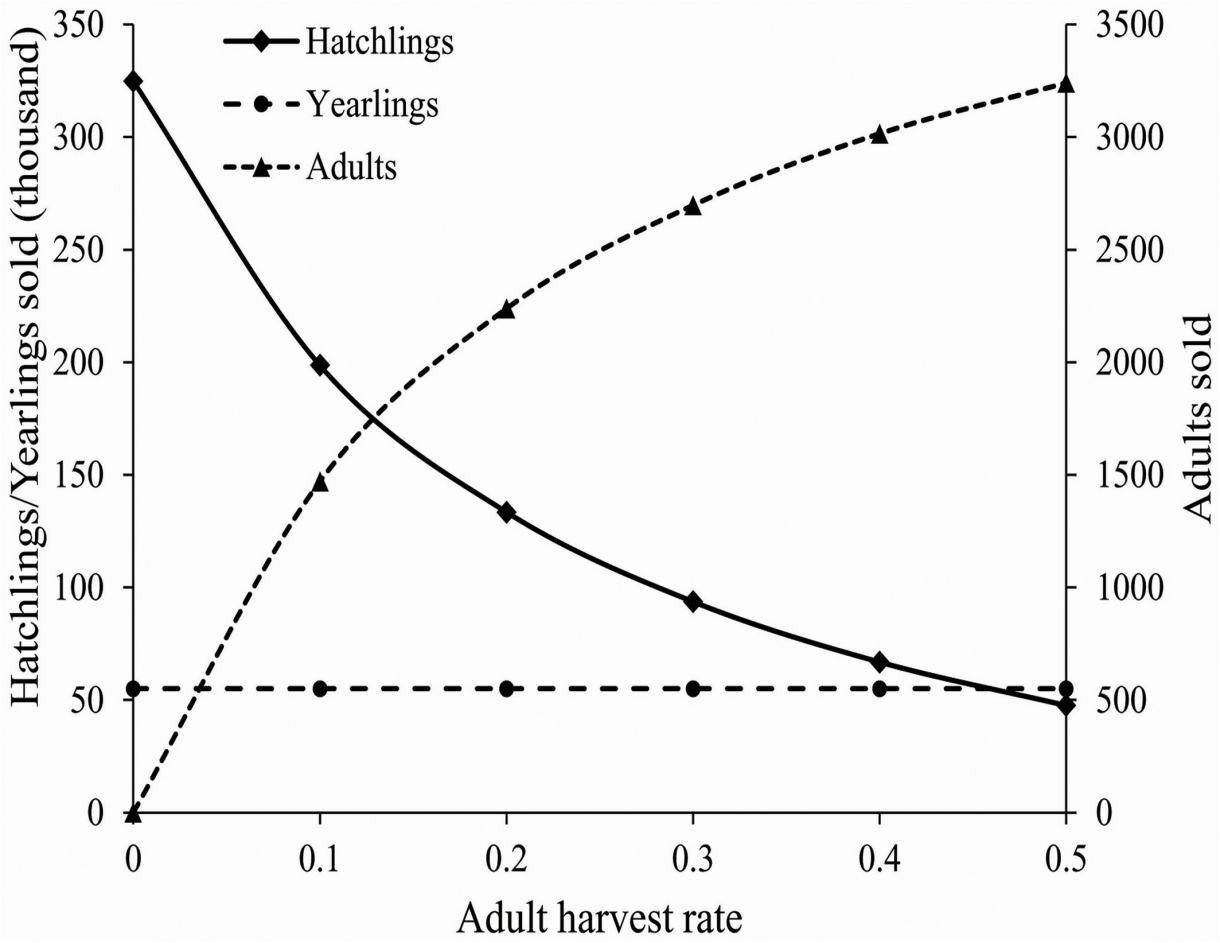
Farming production under different harvest pressures of the adults from the stock, assuming constant production of ~55,000 four inch turtles for domestic markets. As the adult harvest rates increases, the hatchling production gradually decreases.

Under traditional farming, assuming no mass die-offs and average survivorship and per capita birth rates, the model shows annual production of approximately 325,000 hatchlings and 55,000 yearlings, which is in close agreement with actual production based on our interviews with the farm owners. In order for the farm to operate sustainably, approximately 182 male and 546 female head-starts must be released into the stock ponds annually. We incorporated harvest of the adult stock at the following rates: 0.1, 0.2, 0.3, 0.4, and 0.5, to determine its effects on the number of adults, yearlings, and hatchlings sold annually. Depending on the adult harvest rates, 531‒963 male and 1612‒2887 female head-starts should be released into the stock ponds, annually (S1 Table). As expected, harvesting the adult portion of the stock population will decrease the egg production and therefore decrease profit made from hatchling exports. Therefore, loss of profit should be offset by profit made from selling adults, which varies between 1470 and 3240 total individuals in the case of 0.1 and 0.5 harvest rates, respectfully (Fig 3).

### Economy

**Fig 4:**
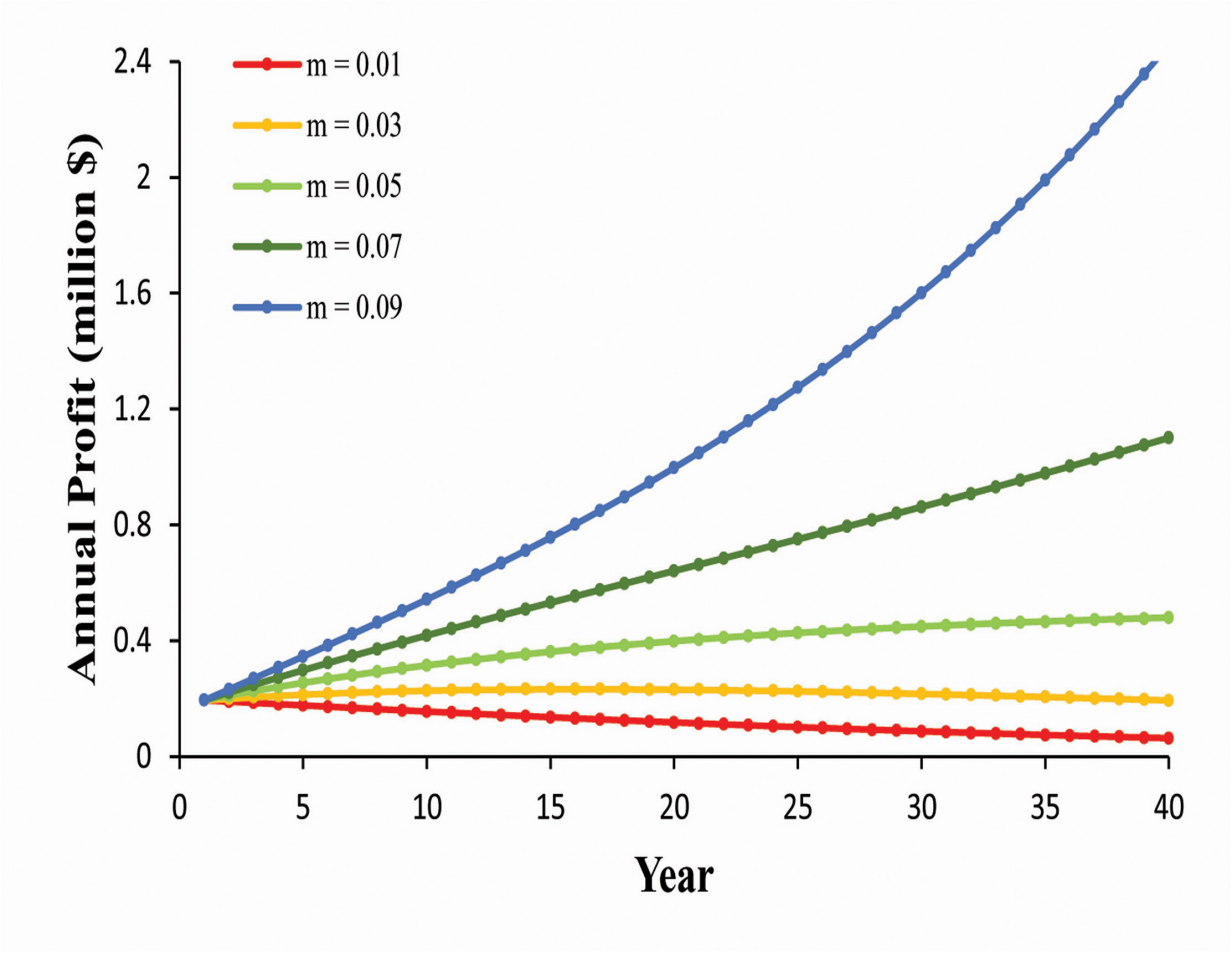
Annual profit from traditional farming operations, assuming discount rate (*d*) of 5% and a range of market rates (*m*) from 1–9%.

Conservative approximation of annual profit under traditional farming (assuming $0.35 price per hatchling and $7.50 price per yearling) is currently ~$194,222. Under 5% discount rate, annual profit will increase only if the market rate is 5% or above (Fig 4). Accumulated total profit over a 40-year period would range between 15.16 and 45.09 million, for 5% and 9% market rates, respectfully. Estimated accumulated profits over a 40-year period, for a range of values of discount and market rates, are presented in Table 3. In the case of 40% adult harvest rate (3,015 individuals/year), only ~66,891 hatchings would be sold, assuming a constant production of ~55,000 yearling turtles. For this operation to meet the profit of ~$194,222, current profit per capita adult turtle would currently have to be ~$13.

**Table 3:** Expected total profit assuming discount rates (*d*) of 3%, 5%, and 7%, and market rates (*m*) of 1%, 3%, 5%, 7%, and 9% accumulated over a 40-year period.

Accumulated profit (million dollars)	*m* = 0.01	*m* = 0.03	*m* = 0.05	*m* = 0.07	*m* = 0.09
*d =* 0.03	6.78	13.20	24.07	43.12	77.45
*d =* 0.05	4.82	8.75	15.16	26.04	45.09
*d =* 0.07	3.60	6.10	10.02	16.44	27.31

### Porter five forces analysis

**Fig 5:**
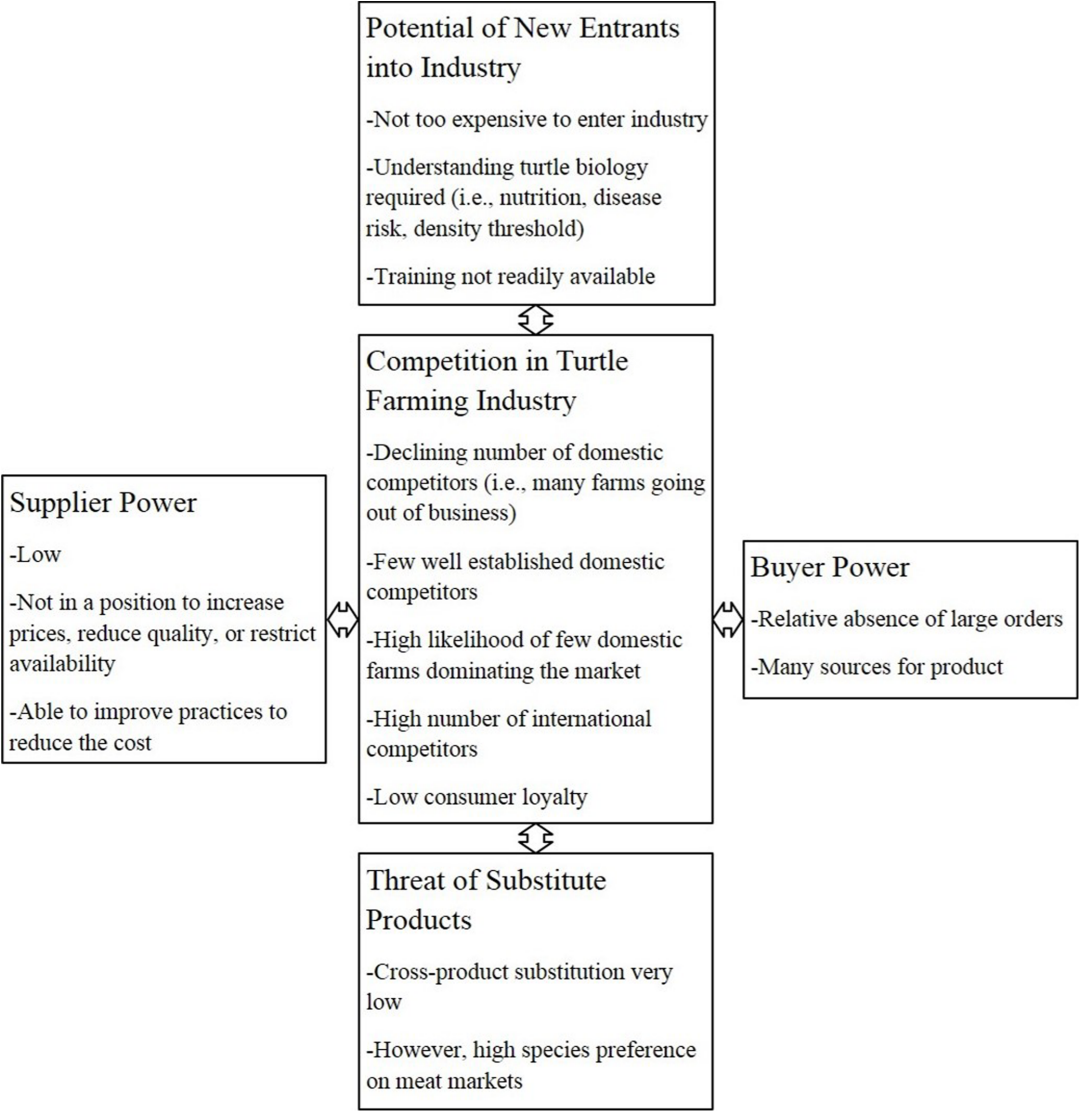
Porter five forces analysis diagram representing underlying drivers of freshwater turtle industry such as the market competition, buyer and supplier power, and threats of substitute products.

Fig 5 represents the output of a Porter five forces analysis. The basic requirements for establishing a turtle farm includes the development of artificial ponds and incubation facilities (“potential of new entrants into industry”). While establishing a freshwater turtle farm does not seem expensive understanding aspects of turtle biology, such as territoriality and disease risk due to overcrowding, is required for the farm to be successful. Such knowledge is usually acquired through trial and error practices rather than training programs.

Over the past two decades competition has been the most dynamic force (“competition in turtle farming industry”) in the turtle farming industry. Significant expansion of farming operations in China led to extreme competition among US farms, resulting in lower prices for many species. As a result many American farms went out of business. Today the industry consists of only a few well established farms that produce hatchlings of less common species and/or four inch turtles.

Products that represent a substitute for turtle meat on Asian markets are essentially nonexistent (“threat of substitute products”). In Asia, turtle meat is not only a protein resource; it is associated with cultural beliefs, such as longevity and healing powers. Therefore, there is very little risk in the turtle farming industry due to substitute products. Similarly, the turtle pet trade remains relatively free of substitution.

Turtle farmers in the US currently have little bargaining power (“supplier power”). A great portion of hatchling turtles used to be exported to supply Asian turtle farms. However, in the past two decades, the Asian farms have become self-sustaining for the more common species and are no longer willing to pay a “break even” price to American farmers for hatchlings. Therefore, US farmers are not in a position to increase prices, reduce quality, or restrict availability for more common species.

The buyer has a great influence especially on large international markets (“buyer power”). For example, the orders from Asia were often on the scale of tens of thousands but today those large orders are much less frequent. In addition, the international buyer has a wide choice between many Chinese and American farms for the more common species. Overall, establishing a new turtle farm appears to be a tough industry to survive in if the focus is on hatchling turtles. The main reason is the competition with well-established domestic and international farms as well as decrease in hatchling turtle demand. However, with a better understanding of effective methods for commercial production of adult turtles where the current demand is high, turtle farming has the potential of remaining a profitable business.

## Discussion

In recent decades, the southern US has played an important role in supplying wild caught freshwater turtles to Asia [14, 15]. To meet demands, turtle farming became a common practice on Asian farms but also a booming aquaculture business in the southern US [16, 19]. Yet, farming practices in the US have been narrowed to commercially raising hatchlings for either the pet trade or to supply Asian turtle farms, which provides no competition to wild turtle collectors. We sought to describe the dynamics of farming operations and economy of traditional vs non-traditional farming by summarizing the cost, price, and demand for turtles in the past, but also by creating predictive models about future cost and revenue. We developed these models in order to explore the possibility of decreasing pressures on wild turtle populations in the US by creating a supply of turtle meat through farming operations. Although, red-eared sliders are currently one of the least desirable species on the meat market (i.e., due to their wide distribution and smaller size), farmers might be forced to shift the production toward red-eared sliders as protein sources if the declines in wild turtle populations persists.

It is important to note that models presented here contain some limitations. Parameters we used were primarily developed based on our interviews with one of the most successful farmers in the US and their staff. We were not able to acquire the necessary information from other turtle farms in the region for two main reasons: 1. industry-level competitive secrecy of farmers toward providing the information and 2. lack of sufficient biological data records on these farms enabling the derivation of model parameter values. The international farms are even more secretive of their farming operations. As one consequence, peer reviewed literature on turtle farming models is not often available although some studies have described certain aspects (i.e., overall production) of turtle farming practices but extracting the metadata provided in that literature was not adequate for model development. In comparison to our model, it is possible that other farms in the region operate under slightly different circumstances (i.e., different feeding practices or artificial pond design), which would in turn affect the productivity as well as the cost of production. Our representative farm invests in motorized machinery for feeding purposes and both stock and green-house pond cleaning, and it is one of the first farms in the US to construct green-house ponds for enhanced growth purposes (Jesse Evans, Concordia Turtle Farm). Second, the farmers remained somewhat reserved for the fear of competition and their practices being implemented on other farms (i.e., we were not allowed to enter the green-houses). Therefore, we were only able to obtain general information on the cost of farming operations (i.e., $0.25/hatchling and $4.5/yearling turtle) while we did not acquire specific cost such as feeding, maintaining the stock pond, building maintenance, etc. Due to necessity to implement such simplifications in the model, our results remain a tentative approximation. In comparison of our model to the real time production data on the farm, our results represent the best case scenarios. For example, the results of traditional farming in our model is achieved on farms during extremely “good years”. One explanation is that we explored farming production to its fullest potential by maintaining the maximum stock density and optimal sex ratio while farmers usually do not strictly control these factors.

Financial analyses showed that in order to make the same profit as created in selling strictly hatchling and yearling red-eared sliders, profit must be minimum of ~$13/turtle, with mean size of adult turtle on the farm of ~1.6 kg. Based on the interviews with meat market establishments, 20–35 lb turtle (probably referring to snapping turtles) provided 8–10 lb of meat [14]. Using the same logic of approximately 40% of red-eared slider weight consisting of meat after processing, the minimum profit requirement is $20.31/kg of meat. To give perspective, we made comparisons of our model requirements to prices of other most often reported species on meat markets. On domestic markets, Ceballos and Fitzgerald (2004) [14] reported the lowest price of $3.00 and highest price of $10.89 per kilogram of turtle meat. Species being sold were not specified in this report but assumed that most were snapping and softshell turtles. Currently, several online sources are selling turtle meat for $55-$66/kg, but again the seller is not specifying the species being sold. Another online source is advertising wild caught snapping turtle meat for ~$44/kg. These North American species are also being farmed in Asia, making an actual source even more unclear. Haitao et al. (2008) [16] interviewed Chinese turtle farms and reported that red-eared sliders were sold for $4.11/turtle or $10/kg and snapping turtles (*Chelydra serpentina*) being sold for $25.24/individual or $45/kg.

Our estimate of profit requirements for adult turtles sold into the meat market seems to exceed current market prices. In addition, Louisiana is one of several states in the southeast US that still allows unlimited take of non-CITES listed freshwater turtles from the wild [15]. Because of high costs associated with commercially producing adult red-eared siders on the farms, commercial harvest of wild adults in unregulated states is likely to continue and even increase due to recently implemented regulations in surrounding states [15], further exacerbating the threat to wild populations. Based on our interview with the farm owners, in Louisiana, wild caught adult red-eared sliders can be purchased for as low as $0.60/turtle, making profitable commercial production of adult red-eared sliders currently unachievable.

Because the current market/demand is more stable for 4 inch red-eared sliders than hatchling turtles, our model operates under the assumption that demand for 4 inch turtles will remain constant and that all hatchling turtles produced will be sold. Price and demand for hatchling turtles that are exported overseas have varied in past years (Jesse Evans, Concordia Turtle Farm). Louisiana Department of Agriculture and farm owners speculate that the decrease in demand is due to Asian farms becoming self-sustaining and Asian farmers now exporting hatchlings for the pet trade. Competition within the US also plays an important role. In the early 2000s, when popularity and demand for turtles on foreign markets was high, many farms were established in Louisiana, which might have led to the subsequent decrease in hatchling prices [22]. In more recent years, turtle farming operations have become less numerous [22] (Jesse Evans, Concordia Turtle Farm). We speculate that the reason for the current rise in price of hatchlings is due to a loss of supply. However, decreasing demand for hatchling red-eared sliders and also future declines in wild populations may shift farm operations towards producing larger turtles, especially for more desirable species. Additionally, a shift could also occur under government programmatic support for raising threatened species hatchlings and head-starts as an alternative source of revenue for turtle farmers.

Such a shift in production goals could also be enabled by decreasing the cost of farming operations. The cost of maintaining green-houses and stock is a crucial part of making a profit from adult turtles. The cost of raising head-start red-eared sliders is particularly expensive (~$4.50 per turtle) mainly due to the costs associated with heating greenhouse ponds during winter months. However, future improvements and changing how farms are maintained can lower costs. For example, adding cheap fresh fish (e.g., an invasive species like carp) into the diet and raising corn, instead of using the more expensive commercial pellets. Although not yet implemented, switching to solar energy would decrease the long-term costs of greenhouse maintenance. On the other hand, unpredictable fuel costs for machinery (i.e., for feeding and cleaning the ponds) and inflation rates may impact future costs.

We modeled the dynamics of turtle farming using one of the most common species of freshwater turtles in the wild and one of most commonly and successfully farmed species in the US. Red-eared sliders also represent ~50% of all turtles on the farm while the rest is a mix of other species such as yellow-bellied sliders (*Trachemys scripta scripta*), snapping turtles (*Chelydra serpentina*), softshell turtles (*Apalone sp*.), etc. Although harvesting adult red-eared sliders from stock is not profitable under current market conditions, this model can now be modified to fit other species that are currently more desirable for the meat markets. For example, snapping and softshell turtles are desirable on the Asian meat markets and have higher values as meat than do red-eared sliders, but currently these taxa are only exported as hatchlings. We are interested in continuing to modify the existing model when more knowledge is acquired about the life histories and space requirements for these other species, and we intend to model the potential of raising snapping and softshell turtles to adulthood and selling them directly to the meat markets when these data become available.

## Supporting Information

S1 TableNumber of hatchling *H*(*t*), yearling *Y*(*t*), and adult *A*(*t*) turtles produced for profit under different adult harvest rates ${(h}_{f}^{F/M})$.Hatchling harvest rate ${(h}_{0}^{F})$ and birth adjustment rate (ADJ) were actively controlled in the model in order to produce ~55,000 yearlings for profit (*Y*(*t*)) and maintain optimal 0.33 adult sex ratio. Number of head-starts released to the stck, $G_{1}^{F}(t)$ and $G_{1}^{M}(t)$, was modeled to meet the maximum stock density allowed.(PDF)

## References

[1] Halliday TR. The extinction of the passenger pigeon *Ectopistes migratorius* and its relevance to contemporary conservation. Biol Conserv. 1980; 17: 157–162.

[2] Heinsohn R, Lacy RC, Lindenmayer DB, Marsh H, Kwan D, Lawler IR. Unsustainable harvest of dugongs in Torres Strait and Cape York (Australia) waters: two case studies using population viability analysis. Anim Conserv. 2004; 7: 417–425.

[3] Jonzen N, Lundberg P, Cardinale M, Arrhenius F. Variable fishing mortality and the possible commercial extinction of the eastern Baltic cod. Marine Ecology Progress Series. 2001; 210: 291–296.

[4] Klemens MW. Turtle Conservation. Washington, D.C.: Smithsonian Institution Press; 2000.

[5] Alves RR, Santana GG. Use and commercialization of *Podocnemis expansa* (Schweiger 1812) (Testudines: Podocnemididase) for medicinal purposes in two communities in North of Brazil. Journal of Ethnobiology and Ethnomedicine. 2008; 4: 3 10.1186/1746-4269-4-3 18208597 PMC2254592

[6] Chen TH, Chang HC, Lue KY. Unregulated trade in turtle shells for Chinese traditional medicine in East and Southeast Asia: the case of Taiwan. Chelonian Conserv Biol. 2009; 8: 11–18.

[7] Fordham DA, Georges A, Brook BW. Indigenous harvest, exotic pig predation and local persistence of a long‐lived vertebrate: managing a tropical freshwater turtle for sustainability and conservation. J of Appl Ecol. 2007; 45: 52–62.

[8] Brown DJ, Farallo VR, Dixon JR, Baccus JT, Simpson TR, Forstner MRJ. Freshwater turtle conservation in Texas harvest effects and efficacy of the current management regime. J Wildl Manage. 2011; 75: 486–494.

[9] Eisemberg CC, Rose M, Yaru B, Georges A. Demonstrating decline of an iconic species under sustained indigenous harvest–The pig-nosed turtle (*Carettochelys insculpta*) in Papua New Guinea. Biol Conserv. 2011; 144: 2282–2288.

[10] Zimmer-Shaffer SA, Briggler JT, Millspaugh JJ. Modeling the effects of commercial harvest on population growth of river turtles. Chelonian Conserv Biol. 2014; 13: 227–236.

[11] Cheung SM, Dudgeon S. Quantifying the Asian turtle crisis: market surveys in southern China, 2000–2003. Aquat Conserv Mar Freshw Ecosys. 2006; 16: 751–770.

[12] Gong SP, Chow AT, Fong JJ, Shi HT. The chelonian trade in the largest pet market in China: scale, scope and impact on turtle conservation. Oryx. 2009; 43: 213–216.

[13] Xianlin M, Zhihua Z, Stuart BL. Recent actions by the People’s Republic of China to better control international trade of turtles. Turtle and Tortoise Newsletter. 2002; 5: 15–16.

[14] Ceballos CP, Fitzgerald LA. The trade in native and exotic turtles in Texas. Wildl Soc Bull. 2004; 323: 881–891.

[15] Mali I, Vandewege MW, Davis SK, Forstner MRJ. Magnitude of freshwater turtle exports from the US: long term trends and early effects of newly implemented harvest management regimes. PLoS One. 2014; 9(1). 10.1371/journal.pone.0086478 24475128 PMC3903576

[16] Haitao S, Parham JF, Zhiyong F, Meiling H, Feng Y. Short communication evidence for the massive scale of turtle farming in China. Oryx. 2008; 42: 147–150.

[17] Haitao S, Parham JF, Lau M, Tien-His C. Farming endangered turtles to extinction in China. Conserv Biol. 2007; 21: 5–6. 17298500 10.1111/j.1523-1739.2006.00622_2.x

[18] Pongtanapanich T. Economic analysis of production and marketing of soft-shell turtle (*Trionyx sinensis*). Witthayasan Kasetsart. 2007; 22: 42–54.

[19] Hughes DW. The contribution of the pet turtle industry to the Louisiana economy. Aquaculture Economics and Management. 1999; 3: 250–214.

[20] Close LM, Seigel RA. Differences in body size among populations of red-eared sliders (*Trachemys scripta elegans*) subjected to different levels of harvesting. Chelonian Conserv and Biol. 1997; 2: 563–566.

[21] Harris JR, Neil KP, Behravesh CB, Sotir MJ, Angulo FJ. Recent multistate outbreaks of human *Salmonella* infections acquired from turtles: a continuing public health challenge. Clin Infect Dis. 2010; 50: 554–559. 10.1086/649932 20085463

[22] Louisiana State University AgCenter. Agriculture is backbone of Louisiana’s economy. 2013; Available: http://www.lsuagcenter.com/agsummary/home.

[23] Eagle J, Naylor R, Smith W. Why farm salmon outcompete fishery salmon. Mar Policy. 2003; 28: 259–270.

[24] Heykoop J, Frechette DL. Gatornomics: profitable and sustainable use of alligators in Southeastern United States. Mar Resour Econ. 2001; 16: 127–142.

[25] Porter ME. Competitive strategy: techniques for analyzing industries and competitors Simon and Schuster; 2008.

[26] Ernst CH, Lovich JE. Turtles of the United States and Canada. 2nd ed. Maryland: The John Hopkins University Press; 2009.

[27] Nijman V, Shepherd CR. Trade in non-native, CITES-listed, wildlife in Asia, as exemplified by the trade in freshwater turtles and tortoises (Chelonidae) in Thailand. Contrib Zool. 2007; 76: 207–212.

[28] Ewert MA, Jackson DR, Nelson CE. Patterns of temperature-dependent sex determination in turtles. J Exp Zool. 1994; 270: 3–15.

[29] Wibbels T, Cowan J, LeBoeuf R. Temperature-dependent sex determination in the red-eared slider turtle, *Trachemys scripta* . J Exp Zool. 1998; 28: 409–416. 10.1002/(sici)1097-010x(19980801)281:5<409::aid-jez6>3.0.co;2-s 9662828

