# Supplementary material for: Modeling Commercial Freshwater Turtle Production on US Farms for Pet and Meat Markets
Source: PLoS One. 2015 Sep 25;10(9):e0139053. doi: 10.1371/journal.pone.0139053 (PMC4583382; doi:10.1371/journal.pone.0139053)
Supplement: S1 Table — Hatchling harvest rate (h0F) and birth adjustment rate (ADJ) were actively controlled in the model in order to produce ~55,000 yearlings for profit (Y(t)) and maintain optimal 0.33 adult sex ratio. Number of head-starts released to the stck, G1F(t) and G1M(t), was modeled to meet the maximum stock density allowed. (PDF) [file pone.0139053.s001.pdf]

h_0^F	ADJ	$H(t)$	$G_1^F(t)$	$G_1^M(t)$	$Y(t)$	$h_f^{F/M}$	$A(t)$
0.7489	0.99765	198,655	1,612	531	55,005	0.1	1,470
0.66496	0.99578	133,484	2,162	723	55,006	0.2	2,238
0.58075	0.994	93,711	2,498	828	55,005	0.3	2,697
0.4962	0.99213	66,891	2,724	909	55,004	0.4	3,015
0.41136	0.9903	47,597	2,887	963	55,005	0.5	3,241
